# Is It Ethical to Use Enhancement Technologies to Make Us Better than Well?

**DOI:** 10.1371/journal.pmed.0010052

**Published:** 2004-12-28

**Authors:** Arthur Caplan, Carl Elliott

## Abstract

Background to the debate: A variety of biomedical technologies are being developed that can be used for purposes other than treating disease. Such “enhancement technologies” can be used to improve our appearance and regulate our emotions, with the goal of feeling “better than well.” While these technologies can help people adapt to their rapidly changing lifestyles, their use raises important ethical issues.

## Abstract 2

Biomedical "enhancement technologies" are being developed that can be used for purposes other than treating disease, such as improving our appearance and regulating our emotions

## Arthur Caplan's Viewpoint: Nobody Is Perfect—But Why Not Try to Be Better?

Perfection has come in for a lot of bad press recently. A torrent of books and articles has recently appeared [1,2,3,4,5,6,7,8,9], all raising serious ethical questions about the wisdom and morality of trying to use biomedical knowledge to perfect ourselves or our offspring.

Biomedical scientists and physicians might be inclined to ignore this literature as just so much abstract philosophical handwringing. After all, it is almost impossible to find mainstream scientists arrogant enough to proclaim their interest in perfecting anything, much less themselves or their fellow human beings.

Beating up on the pursuit of perfection is silly. As Salvadore Dali famously pointed out, “Have no fear of perfection—you'll never reach it.” Critics of those who allegedly seek to perfect human beings know this. While often couching their critiques in language that assails the pursuit of perfection, what they really are attacking is the far more oft-expressed—albeit far less lofty—desire to improve or enhance a particular behavior or trait by the application of emerging biomedical knowledge in genetics, neuroscience, pharmacology, and physiology. Those who might accurately be termed “anti-meliorists” wonder how we will ever resist the obvious temptation to put this knowledge to use to alter ourselves. They are quick to note that we have already given in to such temptation—we augment our breasts, smooth our wrinkles, and pump ourselves full of antidepressants.[Fig pmed-0010052-g001]

**Figure pmed-0010052-g001:**
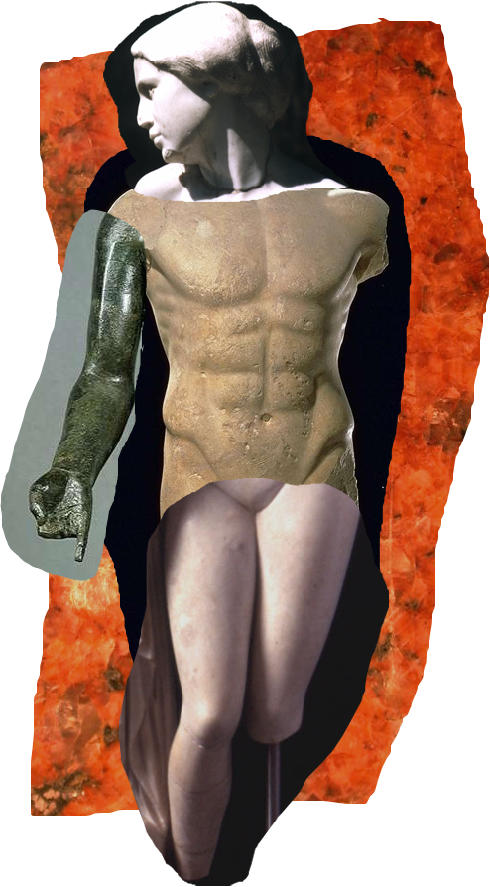
It is in our nature as humans to strive for self-improvement (Illustration: Margaret Shear)

Putting the brakes on biologically driven human betterment would have real consequences for science. Some lines of research would be slowed or restricted [3,5,8]. Their application would be declared off-limits or at least tightly regulated [1,2,3,4,5,7,8,9].

Why is the drive to improve ourselves so disturbing to the anti-meliorists? Their arguments cluster around three key worries: that the pursuit of perfection by biomedical means is vain, selfish, and unrewarding [1,2,3,6,7], that improving ourselves is unfair [1,3,4], and that enhancement or improvement violates human nature [2,4,5,7,8,9] and may actually destroy it [2,5,7,9]. It is the last of these arguments that is at the core of anti-meliorist concerns.

It cannot simply be the pursuit of improvement that is making anti-meliorists nervous. Many religious traditions and spiritual movements seek perfection [10,11,12,13], but these evoke no negative commentary from the anti-meliorists. Nor do efforts to improve animals and plants set this crowd aflutter. Rather, it is biomedical knowledge being applied to you and me that is the crux of their concern. They fear that in applying new biomedical knowledge to improve human beings, something essential about humanity will be lost. If biomedical tinkering is allowed, we will destroy the very thing that makes us human—our nature.

Anti-meliorism rests, however, on a very shaky foundation. To support their position, the anti-meliorists must state what human nature is. They do not. They must also be very clear about why they see human nature as static. They are not. And they must advance an argument about why human nature, which has presumably evolved in response to an enormous array of random forces, tells us anything about what is good or desirable in terms of the traits humans should possess. They cannot.

The fight over whether there is any such thing as human nature is a long-standing one [14]. But one can concede that we are shaped by a causally powerful set of genetic influences and still remain skeptical as to whether these produce a single “nature” that all members of humanity possess. Is there a single trait or fixed set of traits that defines the nature of who we are and have been throughout our entire existence on this planet? Unless they can articulate this Platonic essence, anti-meliorists do not have a foundation for their argument that change, improvement, and betterment are grave threats to humanity.

Worse still for anti-meliorists, we are clearly creatures who have long tinkered with ourselves, using all manner of technologies from clothing to telescopes to computers to airplanes. Our view of our “nature” is closely linked to the technologies that we have invented and to which we have adapted [15]. We are already technological creatures.

Nor is there any normative guidance offered by our evolutionary history that shows why we should not try to improve upon the biological design with which we are endowed. Augmenting breasts or prolonging erections may be vain and even a waste of scarce resources, but seeking to use our knowledge to enhance our vision, memory, learning skills, immunity, or metabolism is not obviously either.

Ultimately, anti-meliorism posits a static vision of human nature to which the anti-meliorists mandate we reconcile ourselves. If anything is clear about human nature, it is that this is not an accurate view of who we have been or what we are now, or a view that should determine what we become.

## Carl Elliott's Viewpoint: Pharma's Gain May Be Our Loss

Those of us who worry about medical enhancement are usually less worried about the technologies themselves than about the larger social effects of embracing them too enthusiastically. Just as you do not need to object to cars to worry about urban sprawl, you do not need to object to enhancement technologies to question where these technologies may be taking us. It is not just technophobes who wonder whether a society that consumes 90% of the world's supply of methylphenidate (Ritalin), where the most profitable class of drugs is antidepressants, and where cosmetic surgeons perform liposuction on prime-time television is a society that has somehow lost its way.

Let's look at three of the most commercially successful medical enhancements of recent years: selective serotonin reuptake inhibitors, hormone replacement therapy, and the diet drug fenfluramine-phentermine (Fen-Phen). What can we learn from these interventions?

First, the manufacturers of enhancement technologies will usually exploit the blurry line between enhancement and treatment in order to sell drugs. Because enhancement technologies must be prescribed by physicians, drug manufacturers typically market the technologies not as enhancements, but as treatments for newly discovered or under-recognized disorders. Selective serotonin reuptake inhibitors were marketed not as personality enhancers, or even only as treatments for clinical depression, but as treatments for questionable illnesses like “premenstrual dysphoric disorder” [16]. Fen-Phen was sold not as a mere diet drug but as a treatment for obesity, which Wyeth, the manufacturer, portrayed as a dangerous public health problem [17]. Estrogen replacement therapy was initially marketed as a risk-free way for women to extend their youthfulness. But when a 1974 study found that estrogen replacement therapy was associated with an increased risk of endometrial cancer, the manufacturers added progesterone, renamed the combination “hormone” replacement therapy, and recast it as a treatment for medical problems associated with menopause such as osteoporosis [6].[Fig pmed-0010052-g002]

**Figure pmed-0010052-g002:**
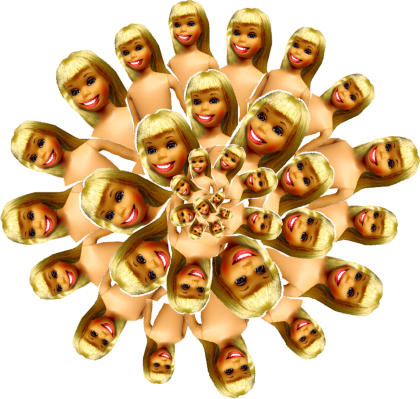
Where is the pursuit of the perfect face, body, and mind taking us? (Illustration: Margaret Shear)

Second, an alarming number of supposedly risk-free enhancements have later been associated with unanticipated side effects, some of them deadly. Wyeth has set aside over $16 billion to compensate the thousands of patients who have developed valvular heart disease and pulmonary hypertension after taking Fen-Phen [18]. A 2002 National Institutes of Health study found that hormone replacement therapy was associated with such an elevated risk of heart disease, stroke, pulmonary emboli, and breast cancer that the study was stopped prematurely [19]. Selective serotonin reuptake inhibitors are currently embroiled in controversy over whether they are associated with an elevated risk of suicide [20].

Third, the most successful enhancement technologies have been backed by tremendously influential public relations campaigns. These campaigns have included ghostwritten journal articles, industry-funded front groups, and lucrative payments to academics, professional societies, and university centers [21]. For example, GlaxoSmithKline marketed paroxetine (Paxil) by promoting the previously obscure diagnosis of “social anxiety disorder” through phony support groups, celebrity spokespeople, a direct-to-consumer illness awareness campaign, and generous payments to key opinion leaders [22]. The manufacturers of estrogen replacement therapy marketed the hormone in the 1960s by funding a “research foundation” for Robert Wilson, the gynecologist and author of the best-selling book *Feminine Forever* [6]. Wyeth marketed Fen-Phen by funding obesity research centers, launching public fitness campaigns, contracting with a medical education company to produce a series of ghostwritten journal articles, and making generous payments to academic physicians who then published extensively and testified for the drug's safety to the Food and Drug Administration [17].

The traditional worry about enhancement technologies is that users of the technologies are buying individual well-being at the expense of some larger social good. I may improve my own athletic ability by taking steroids, but I set off a steroid arms race that destroys my sport. I may get cosmetic surgery for my “Asian eyes” or use skin lighteners for my dark skin, but I reinforce the implicitly racist social norms that say that Asian eyes or dark skin are traits to be ashamed of. The worry is that some aspect of the way we live together, collectively, is going to be damaged by actions that we take individually [4].

A market-driven health-care system brings this worry much closer to home. The pharmaceutical industry is now the most profitable and politically powerful industry in the United States [23]. It also has a huge financial interest in creating a demand for enhancement technologies. The pharmaceutical industry can buy politicians to pass industry-friendly legislation; it can buy academic scientists to publish favorable journals articles; it can buy professional societies and patient support groups to spread the word on the newly medicalized disorders that its interventions are developed to treat [24]. It can even buy bioethicists to dispense with any moral concerns [25]. In this kind of political and economic climate, how likely is it that dissenting voices will have any effect before it is too late?

## Caplan's Response to Elliott's Viewpoint

Elliott professes to be unhappy about enhancement. What arguments does he present to support his unhappiness? Not many, and the arguments that he does offer miss the point completely.

If people want to feel better, sleep less, have fewer hot flashes, better vision, or fewer wrinkles, then they may want to use enhancement technologies to achieve these things. Technology in itself isn't driving us in any particular direction—I believe that we decide where it should go. Elliott, however, gravely warns us that you and I do not really decide a direction when it comes to matters of enhancement. It is—listen carefully for the Darth Vader–esque hissing—drug companies!

The rest of Elliott's viewpoint amounts to what is his increasingly familiar harangue against the pharmaceutical industry. The drug companies sucker us into buying enhancement by getting us hooked on pseudotherapies. The drug companies rob us of our will to fend off their siren-like messages of better living through their chemistry. And the drug companies get us feeling so bad about ourselves that we empty our wallets on their latest overpriced geegaws.

Pharmaceutical companies may be evil incarnate. And we may be putty in their pecuniary little hands. But that has nothing at all to do with the question of whether there is anything wrong with pursuing enhancement. When Elliott eagerly dons his hair shirt to bemoan Big Pharma, he finds so much sin to revel in that he forgets to give a reason, any reason, why enhancement is, in itself, immoral.

At most he presents an argument for keeping the pharmaceutical industry out of enhancement. Okay, so let's take Big Pharma out of the picture. If we left the encouragement of enhancement to the government, the military, schools, foundations, doctors, or parents, would this now be morally acceptable? I think sometimes it would be. And nothing that Elliott says provides any reason to think otherwise.

## Elliott's Response to Caplan's Viewpoint

Caplan does not defend medical enhancement so much as attack its critics. Or rather, he attacks a small group of conservative critics who want to preserve “human nature.” He dispatches those critics with admirable precision, but I am not sure why he believes that group of critics includes me. My worry about enhancement technologies has little to do with human nature. My worry is that we will ignore important human needs at the expense of frivolous human desires; that dominant social norms will crowd out those of the minority; that the self-improvement agenda will be set not by individuals, but by powerful corporate interests; and that in the pursuit of betterment, we will actually make ourselves worse off.

It's no secret that many Americans are deeply ashamed of their personal shortcomings and inadequacies. Nor is it any secret that these shortcomings and inadequacies can be exploited for commercial profit. But do we really want to submit our health-care system to the same forces that have made millionaires out of motivational speakers and diet book authors?

Skepticism about enhancement technologies is not equivalent to a wish to set back medical research and declare some applications off-limits. This is a debate about enhancing human traits, not curing human illness. To say that our medical research agenda will be set back if we restrict enhancement technologies makes no more sense than saying that cancer surgery will be set back if the American Broadcasting Corporation cancels its cosmetic surgery reality TV show *Extreme Makeover*.

We live in a country where 46 million uninsured people cannot get basic medical care, while the rest of us spend a billion dollars a year on baldness remedies. It is not just the inequity here that is so impressive. It is the fact that we have gotten so accustomed to the inequity that we do not see it as obscene.

## Abbreviation

Fen-Phen: fenfluramine-phentermine
